# The use of real-world evidence among healthcare payers: a scoping review

**DOI:** 10.1017/S0266462325100445

**Published:** 2025-09-12

**Authors:** Lisa Masucci, Diedron Lewis, Jiahao Zhao, Caitlin Carter, Kelvin K.W. Chan, William W.L. Wong

**Affiliations:** 1 Toronto Health Economics and Technology Assessment Collaborative, Toronto General Hospital, Toronto, ON, Canada; 2 School of Pharmacy, University of Waterloo, Waterloo, ON, Canada; 3 Sunnybrook Health Sciences, Centre Odette Cancer Centre, Toronto, ON, Canada

**Keywords:** real-world evidence, payer decision-making, funding arrangements, real-world data, payers

## Abstract

**Introduction:**

Real-world evidence (RWE) is increasingly used to assess and make regulatory decisions on health technologies. However, its application in healthcare payer decision-making is less well-known.

**Objectives:**

The objectives of this study were to (i) review the recent literature on how RWE has been used by healthcare payers, (ii) highlight barriers that limit the use of RWE in payer decision making, and (iii) explore how RWE has been used in various funding arrangements between payers and manufacturers. The benefits of utilizing RWE are also discussed.

**Methods:**

A scoping review was conducted on articles published between 2014 and 2025 in PubMed (Medline), OVID EMBASE, Cochrane Library, and ProQuest Dissertations and Theses Global. Eligible articles were those written in English that discussed the use of real-world evidence among healthcare payers/decision-makers for health technology reimbursement decisions.

**Results:**

Nineteen articles were selected for full-text review based on the inclusion criteria. The review highlighted payers’ interest in incorporating RWE into funding and reimbursement decisions to address uncertainty in the performance of new health technologies. However, a lack of standards for collecting, analyzing, and reporting RWE limits its use. Little is known about how RWE is used in reimbursement decisions since contractual arrangements between payers and manufacturers are confidential.

**Conclusions:**

Although payers are interested in using RWE to inform funding and reimbursement decisions, there are concerns regarding the scientific rigor used to generate such evidence. Having more insight into the contractual arrangements between payers and manufacturers would help to better understand how RWE informs these agreements.

## Introduction

Healthcare payers face challenges in deciding which health technologies to fund, given a limited budget (1–3). This challenge is magnified given the high volume of new treatments seeking timely regulatory and reimbursement approvals, the rising cost of these technologies, and the growing demand for access to effective and innovative treatments and services (1;2;4;5). With increased pressure to make decisions based on limited or single-arm trial evidence, payers are cautious about which technologies to fund (1;6).

Randomized controlled trials (RCTs) are the gold standard for generating scientifically grounded evidence (7;8). However, RCTs seldom reflect conditions in the real world because they tend to have strict inclusion and exclusion patient criteria (3;8–10), lack external validity/generalizability, use intermediate outcomes, and have short-term follow-up (1;3;10). There are also practical and ethical concerns to conducting longer-term studies, as RCTs are often costly to execute, and patients may be unwilling to undergo randomization if the performance of a product is already known (3). With these limitations, funding decisions are made with a great deal of uncertainty as decision-makers operate with imperfect information (3;11;12).

Real-world evidence (RWE), which is derived from sources outside of RCTs, can be used by payers and health technology assessment (HTA) agencies to inform decisions regarding comparative effectiveness, cost-effectiveness, safety, and the overall value of a healthcare technology (3;13–16). Increasingly, RWE is recommended and used to supplement evidence from RCTs to support coverage decisions (17). For payers, RWE on the clinical validity and utility of a particular technology can be used in pre- and post-market evaluations to inform price negotiations and future funding/reimbursement arrangements with manufacturers (14;18–25).

Although there are potential benefits to incorporating RWE in HTA and payer decision making, few countries have established the policy infrastructure and frameworks needed to realize these benefits (3). Many countries are still grappling with how best to include RWE in healthcare decision-making frameworks because the methods of data collection and analysis are not standardized (6;25;26). Although HTA agencies and other multistakeholder groups have provided frameworks to facilitate the adoption of RWE in healthcare decision making (27), the application of these frameworks by payers is still not well understood. For these reasons, data from RCTs are used as the principal source of evidence to inform pre- and post-market funding decisions and pricing arrangements, despite the growing interest in the use of RWE among payers (3;17). Incorporating RWE into funding and pricing decisions can facilitate the establishment of more innovative funding arrangements and incentivize manufacturers to bring new products to the market (25;28).

The primary objective of this scoping review is to summarize the existing literature on how RWE has been used by healthcare payers by (i) identifying the types of RWE healthcare payers have considered, (ii) highlighting the barriers that limit the use of RWE in payer decision making, and (iii) exploring how RWE has been factored into payer/manufacturer funding arrangements. This review focused on both medical devices and medicines.

## Methods

This scoping review was conducted based on the five-step framework proposed by Arksey and O’Malley (2005), that was later updated by Levac et al. (2010), the Joanna Briggs Institute (2015), and Peters et al. (2020) (29–31). Reporting was done according to the Preferred Reporting Items for Systematic Reviews and Meta-analysis for Scoping Reviews (PRISMA-ScR) guidelines (32;33). The protocol for this scoping review is available on the Open Science Framework (https://osf.io/8wpnb/).

### Search strategy

A literature search was conducted by an information specialist (CC) and included literature published between January 1, 2014, and March 26, 2025. The publication date range was selected as this timeframe aligns with the development of the discourse on RWE in HTA and payer decision-making. The databases searched included PubMed (Medline), OVID Embase, Cochrane Library, and ProQuest Dissertations and Theses Global. The first three databases were chosen based on recommendations from Cochrane (34). The last database was chosen as it included dissertations that may lead to published sources. The search strategy included a combination of keywords (limited to title and abstract fields only) and controlled vocabulary (dependent on database availability) related to the concepts of RWE, payers, and decision making. The complete search strategies for each database are provided in Supplementary File 1. The reference lists of included studies were scanned to identify additional relevant studies. All database search results were imported into Covidence (Veritas Health Innovation) for screening.

Two reviewers (JZ and DL) screened the titles, abstracts, and relevant full-text articles independently. Before screening, both reviewers pilot-tested the record screening with a random sample of 50 records. Screening commenced when at least 75 percent agreement was reached. Any discrepancies were discussed between reviewers until a consensus was reached. Disagreements were resolved by a third reviewer (LM). Following the title/abstract screening, only relevant articles were selected for full-text review by the reviewers (JZ and DL).

### Eligibility criteria

Eligible articles were those that (i) discussed the use of RWE among healthcare payers/decision-makers for health technology reimbursement decisions or (ii) discussed the use of RWE in innovative funding arrangements. Studies could explicitly mention RWE or the use of data obtained outside of randomized-controlled trials. Only articles written in English were included. Conference abstracts were removed from the results of all databases. No restriction was placed on the type of healthcare technology included (e.g., medical device or medicine) because it was anticipated that the literature did not sufficiently address any one type of health technology. A PRISMA flow chart (32) was generated to show the outcome of the study selection process (see Supplementary Figure S1).

### Types of sources

This scoping review considered qualitative, quantitative, or mixed-methods articles as we sought to obtain a discourse around the use of RWE.

### Data charting and synthesis

Data were extracted for all relevant articles by one author, with a second author checking for accuracy. Data were extracted and stored in a customized data abstraction form created in Microsoft Excel. The following variables were extracted: disease area, definition of RWE, reason for using RWE, type of RWE used, strengths and weaknesses of RWE as identified by healthcare payers, whether RWE was used in any funding arrangements, and what type of funding arrangement was used. Data synthesis was informed by the data analysis recommendations proposed by Pollock et al (35). This included a narrative account of the results of the review and the use of descriptive statistics, such as counts and proportions, to present the results.

## Results

The literature search generated 13,655 articles from the respective databases (OVID Embase: 6,887, PubMed: 5,159, ProQuest Dissertations and Theses Global: 832, Cochrane Library: 777). A total of 4,024 duplicates were removed, and a further 9,584 articles were excluded at the title/abstract screening stage because they did not meet the inclusion criteria. Following the full-text review, nineteen of the remaining forty-seven articles were considered relevant for further analysis. The reasons for excluding articles at the full-text screening stage are included in Supplementary Figure S1. Supplementary Tables S1 and S2 present the data extracted from the studies identified in this scoping review.

### Overview of included studies

#### Study countries

The general characteristics of the studies included in this review are summarized in Table 1. The majority of studies were based on payers from North America; five studies each from both the United States and Canada (2;3;6;8;15;17;20;28;36;37). Other country-specific studies were on payers from the Netherlands, Germany, Italy, and the Kingdom of Saudi Arabia, respectively (38–41). There were three multicountry studies (7;12;), and two studies were not specific to a country (11;43). All nineteen studies were conducted from the perspectives of payers, with relevance for health technology developers/manufacturers, HTA agencies, patients, and regulatory bodies.

**Table 1.** General characteristics of the included studies

Characteristics	Number (*n* = 19)	Percentage (%)
Country		
Multinational setting	3	17%
Canada	5	26%
United States	5	26%
Netherlands	1	5%
Germany	1	5%
Italy	1	5%
Saudi Arabia	1	5%
Not specific to a country	2	11%
Study design		
Survey/interview	9	47%
Case study	1	5%
Discussion papers with or without literature review	7	37%
Quantitative	2	11%
Clinical setting		
Oncology	6	32%
Pharmaceuticals	4	21%
Medical devices	2	11%
Other new health technologies	2	11%
No specific focus	5	26%

Box 1. Challenges with the use of real-world evidence

- Quality of RWE generated (bias and confounding, incomplete or inaccurate data sets)
- Access to timely and relevant data (legal concerns about data sharing)
- Cost involved in accessing and generating data
- Absence of standards for reporting

#### Study design

Nine studies either surveyed or interviewed payers and other stakeholders to gather their perspectives on the incorporation of RWE into funding and reimbursement arrangements (6;7;12;17;20;28;36;37;42). In these studies, all stakeholders were asked to share their views on the perceived benefits, challenges, enabling factors, and application of RWE.

Two studies surveyed payers (36;42). The first study surveyed 221 payers from the United States and had an additional ten payers discuss the survey results (36). The second study, conducted a survey involving thirty current and former payers from across Europe and the United States, who had experience in the coverage and reimbursement of oncology therapies (42).

Seven studies interviewed stakeholders involved in pharmaceutical pricing and decision making. Clausen et al. (2020) interviewed thirty Canadian stakeholders, including six from pricing negotiation organizations (6). Kovács et al. interviewed twenty-five decision-makers from twenty-two European countries (12). Gray and Kenney involved seventeen stakeholders, seven from payer organizations (28), whereas Hampson et al interviewed nine, including two US payers (20). Timbie et al., included twenty-seven stakeholders, four of whom were payers (17). Husereau et al. held multistakeholder meetings with ninety-one participants, including ten payers (37). Pulini et al. did not report the number of interviewees but identified stakeholders from pharmaceutical and clinical research centers (7).

Five studies were discussion papers supplemented with literature reviews. (7;8;20;39–41). Three studies incorporated a nonsystematic review (7;39;40), one study conducted a systematic literature review (8;20), and one study performed a narrative review (41).

Two studies (15;38) adopted predominantly quantitative methods. The first study assessed whether RWE provided by drug manufacturers influenced the funding recommendations of Canada’s Drug Agency for cancer drugs (15). This study concluded that the RWE generated by manufacturers did little to spur positive funding recommendations, instead raising concerns about data standards and quality (15). The second study estimated an optimal period of no more than two years to collect data from patient registries to inform access with evidence arrangements for the coverage of a colon cancer treatment in the Netherlands (38).

Five studies provided decision-making frameworks or methods on how RWE can be incorporated into payer decision-making and reimbursement schemes (2;3;11;12;40). Two studies were discussion papers presenting a framework (11;40), one study presented a process for developing a RWE framework (3), one was a case study (2), and another utilized a focus group to validate a decision-making tool (12).

#### Clinical setting

Studies focused on several clinical settings where RWE can prove valuable, primarily oncology (*n* = 6) (3;6;15;36;38;42), pharmaceuticals (*n* = 5) (2;7;37;41;43), medical devices (*n* = 2) (12;17), and other new health technologies (*n* = 2) (8;39).

A few studies examined payer perceptions of RWE in oncology funding decisions in the United States and Canada (3;15;36). Two focused on the CanREValue project, which is a stakeholder-led initiative aimed at developing a national framework for generating and using RWE in Canadian cancer drug funding decisions (3;6). Other studies focused on pharmaceuticals and emphasized integrating RWE into standardized decision-making frameworks to inform reimbursement (2;7;41;43). Pulini et al. compared how RWE informs market authorization, pricing, and reimbursement in the United States, the United Kingdom, and France. Two other studies proposed decision-making frameworks, including one using multicriteria decision analysis for Canada’s public health system (2;43). Abu-Shraie et al. demonstrated how RWE can be linked to reimbursement arrangements like risk-sharing agreements, where payment is based on treatment performance (41).

Two studies addressed medical devices (12;17). One developed a decision tool for guiding reimbursement of late technology adoption to address uncertainty in reimbursement for medical devices based on coverage with evidence development agreements (12). The other study conducted interviews to explore industry-wide RWE use, highlighting payer’s concerns about RWE rigor despite its value in supplementing trial data (17). Studies on general health technologies or those that did not have a specific application focused on creating payer decision frameworks and their use in informing reimbursement arrangements (11;12;17;20;39;40).

### Definition of RWE

Twelve of the nineteen studies provided formal definitions for RWE (3;6–8;11;15;17;20;37;38;42;43). Three of these studies reported that RWE refers to evidence on the use of medical products, including the potential risks and benefits, based primarily on patient health data and outcomes (7;15;20). Nine studies highlighted that RWE is generated from the analysis of real-world data (RWD) (3;6–8;11;15;20;37;42). This RWD can be collected either prospectively or retrospectively (8;43). Studies added that such data are not derived from RCT settings (6;7;15;17), and RWD is routinely collected from clinical practice (11;43). Two studies identified the primary sources of RWD as electronic health records (EHRs), claims databases, pragmatic trials, and patient registries (8;15). Smartphones, wearable devices, and survey data were also identified as potential tools for generating RWD (8).

### Reason for using RWE

Studies recognized that although payers rely primarily on evidence generated from RCTs, there is growing interest in incorporating RWE in payer decision-making for health technologies, including pharmaceutical and medical devices (6;8;42). Four studies explicitly stated that RWE complements RCTs by strengthening the evidence that payers need to make funding and formulary decisions (6;8;17;36). Although RCTs demonstrate the efficacy of a technology, they are costly, focus on relatively short timeframes, and target narrowly defined populations, limiting their generalizability (6;8;17;37). In contrast, RWE can address these limitations by using data from routine clinical practice, offering broad patient representation and greater external validity (3;20;38). RWE may also be less costly to produce than evidence from RCTs because data can be collected from existing databases such as clinical registries (3;17). Other studies added that the generation of RWE is not restricted to short study timeframes (3;6;17) and can be used to illustrate the clinical and economic value not captured in trials (3;6;20). Comparative effectiveness, cost analyses, treatment adherence, and patient-reported outcomes were highlighted as critical for payer decision making (6;8;17;36;37). RWE was viewed as valuable when other forms of evidence are not available because of rare indications and small study samples, or when data on key variables are not collected in clinical trials (12;17;37).

### Barriers limiting the use of RWE in payer decision making

Several studies identified key barriers to the use of RWE in payer decision making (Box 1). The most cited concern was the quality of evidence generated (7;8;17;37;43), particularly issues of bias and confounding, noted in seven studies (3;6;8;15;20;38;43). These concerns largely stem from the non-randomized nature of RWE, lack of standardization in study design and analysis, limited transparency, and insufficient expertise to generate and analyze RWE. Reporting, selection, and information biases were also reported in two studies (15;20). As Hampson et al. emphasized, these challenges are evident in observational studies because they are often seen as less robust than RCTs (20).

Data access was another major barrier, cited in seven studies (6–8;17;20;37;43). Four studies highlighted that legal and privacy constraints, especially regarding patient-identified data, limited timely data sharing (7;8;20;37). Additionally, it can take 3–7 years to generate prospective RWE and 2–3 years to access it, making it difficult to use up-to-date RWE to inform payer decisions throughout the lifecycle of products (6;43). High costs of data access and generation, especially for establishing registries, were also described as prohibitive (15;36;38).

Other factors highlighted as limiting the use of RWE by payers were incompleteness and inaccuracy in data sets (7;20;43), due to human error in recording data, omission of data points, and gaps in patient’s medical histories (15). Interoperability issues were noted in two studies (17;37), with inconsistencies in outcome definitions and data formats across platforms making it difficult to integrate data sets (37).

Finally, the absence of proper standards of reporting of results raises concerns about potential data mining and undesirable research practices (8;17;20;37). These limitations contribute to payer mistrust of RWE and the continued preference for RCT evidence (6;37).

### How is/was RWE used in healthcare payer/manufacturer funding arrangements

Studies emphasized the growing interest among payers in the use of RWE to make coverage decisions (6;8;36;41). Several studies mentioned that for payers, RWE is specifically considered when making initial coverage decisions at the launch of a health technology and when these decisions are being reassessed, typically after a product has had substantial time in the market (3;6;8;20;36;37). There was consensus that RWE is used to inform coverage, pricing, and reimbursement negotiations between payers and manufacturers (3;6–8;11;15;17;20;37;43). Two studies noted that the initial decision may rely on epidemiological data to define target populations and estimate technology costs (8;36). Several studies reported that RWE also supports reimbursement schemes that address uncertainties around a product’s real-world performance (3;6–8;11;12;36).

Of the ten studies focused on reimbursement schemes, most cited risk-sharing agreements (RSAs)/managed entry agreements (MEAs) as the principal type of coverage schemes (6;7;11;12;17;36;38–41). Three studies pointed out that outcome-based agreements (OBAs), a subset of MEA, were effective at mitigating financial risk by collecting postlaunch clinical data (28;36;41). Relatedly, coverage with evidence development arrangements that generate RWE under research settings was also noted as desirable MEA (11;12;39). However, financial-based MEA, which focuses on cost containment, is more commonly used compared with OBA because they are easier to implement (41).

Overall, three studies agreed that MEA promotes efficiency and sustainability in the health system by guiding resource allocation and defunding costly, low-value technologies (3;6;37). Another three studies highlighted the challenges around establishing OBA, (28;36;41) including monitoring outcomes, patient adherence tracking, contract complexity and costs, lengthy negotiations, and methodological issues such as endpoint selection, identifying target populations, and appropriate sample sizes for analysis (28;36;41).

In terms of the application of RWE, four studies explained that little is known about its use in payer decision-making and coverage arrangements (especially in oncology) because this information is embedded in confidential contractual agreements between payers and manufacturers that are restricted from public access (28;36;39;40). One study added that only 17 percent of the 99 stakeholder organizations included in their study had experience using RWE in OBA (36).

### Types of RWE used and utility of various forms of RWE

In examining the role of RWE in payer decision-making and contractual arrangements, four studies (28;36;38;39) compared different RWD sources: registry data, claims data, and EHRs. Registry data reflect clinical practices, capture larger populations than RCTs, and can be used to estimate real-world cost and effectiveness (38;39). However, registries are often costly to establish (39), and the evidence is susceptible to bias due to lack of randomization (38). Claims data, typically from insurance schemes, were considered more appropriate for MEA that seeks to mitigate uncertainty around the utilization and cost of new health technologies but are less useful for agreements that require evidence on safety or clinical effectiveness (28;39). It was also argued that claims data are more readily available and less costly than data generated from registries and clinical trials, requiring minimal input from patients and clinicians (28;36;39). For clinical effectiveness and safety, EHR data were preferred by payers because information on clinical variables is likely to be recorded (28;39).

### Framework/tools that incorporate RWE into payer decision-making and reimbursement schemes

Some studies proposed frameworks on how RWE can be incorporated into payer decision-making and reimbursement schemes (11;12;40). One study developed a lifecycle HTA framework that provides guidelines for payers to evaluate new evidence (including RWE) on health technologies through decision rules that are outlined in conditional market access agreements aimed at sharing risk and mitigating uncertainty in product performance (11). Another study also proposed a framework that allows payers and pharmaceutical manufacturers to develop and optimally utilize RWE in funding decisions (43). This framework is grounded in the principles of transparency, communication, and collaboration among stakeholders, which in turn influence the process of developing RWE. Using a similar approach, a third study proposed a new pathway for value-based managed entry agreements using the existing pricing and reimbursement mechanism (40). They argued that this pathway can be adapted to the health systems in other countries with special consideration for factors such as the details of the MEA and the process of evidence generation (40).

## Discussion

This review provided a narrative synthesis of how RWE has been used by healthcare payers, the barriers to use, and how RWE is currently used in funding arrangements. It highlighted the types of RWE considered by payers and described how RWE has been factored into funding arrangements. The review revealed payers’ interest in incorporating RWE into coverage decisions and funding arrangements for new health technologies, especially for cancer drugs and other pharmaceutical and medical devices (6;8;17).

Payers agree that RWE can supplement evidence from RCTs to build the evidentiary case for reimbursing health technologies, where there is uncertainty around long-term clinical effectiveness, safety, and real-world cost-effectiveness (6;8;17;36). RWE is also preferred when other forms of evidence are not available because of rare disease indications, small study samples, or when data on key variables are not collected in clinical trials. RWE studies are particularly attractive because evidence generated is likely to result in generalizability (3;6;8;17).

The literature explained that the use of RWE in payer decision-making is critical at two junctures: (i) when making initial funding decisions and (ii) when reassessing initial funding decisions. Funding arrangements are often in the form of managed entry agreements that allow conditional access to new health technologies, as RWE around the cost and effectiveness is collected and evaluated (39;40). The goal of these agreements is to manage uncertainty through risk-sharing between payers and technology developers (39).

Healthcare payers also acknowledged the many barriers that may limit the use of RWE in payer decision making. These barriers stem from the lack of standard scientific methods of data collection, analysis, and reporting of RWE (6;7;15;17;20;37). These challenges give rise to concerns of bias and confounding, making RWE less reliable for scientific research and payer decision-making. They also justify the preference for RCT evidence in payer decision-making (6). Legal barriers around data privacy and data access also limit the availability of RWE for decision-makers (7;8;20;37).

To address these challenges, payers have called for greater collaboration with manufacturers in the generation and use of RWE (43). Standardizing methods of data collection and analysis are desirable to ensure that RWE is trustworthy (20). Researchers have also proposed decision frameworks that utilize RWE in payer coverage decision-making throughout the lifecycle of new health technologies (11;43). These frameworks provide a structured approach to generating and assessing RWE in payer decision-making (11;43). Although the literature provides several recommendations on conducting and reporting RWE (44–47), researchers have reported a lack of consensus on these guidelines (20). This highlights the need to consolidate and standardize RWE research and reporting practices to better serve various stakeholders, including payers.

An important finding of this scoping review is that published examples of how RWE is incorporated into payer decision-making and funding arrangements are not readily available. This is because contractual agreements between payers and health technology developers are often confidential and not publicly available (28;36;39;40). As a result, stakeholders such as payers, manufacturers, and researchers have limited information on how best to incorporate RWE in decision-making and coverage arrangements. Options for disseminating successful cases that do not violate contractual agreements should be explored. This scoping review highlighted the usefulness of certain data sources for generating RWE, including registries, insurance claims, and EHRs. However, the included studies cautioned that payers should be mindful of the advantages and limitations of each source of data.

The results of this scoping review are consistent with a previous US-based review that examined the use of RWE in payer decision-making, with a specific focus on next-generation sequencing tests (25). The review highlighted the value of RWE in outcomes-based contracts, the importance of data sharing, integrating clinical and genomic data, and the need for regulatory and practical solutions to support the use of RWE (25). However, the narrow focus on a single country and clinical intervention limits its scope, whereas this current review adopts a much broader focus and is not restricted to a particular country or health intervention.

There are a few limitations of this study. First, a risk-of-bias assessment to evaluate the quality of the included studies in this scoping review was not conducted. A risk-of-bias assessment is not typically conducted for scoping reviews, so the authors cannot make a definitive statement on the quality of the included articles. Second, many of the included studies were qualitative with small sample sizes and specific to a region. The individual study findings may not be generalizable, but together they are valuable in summarizing the use of RWE among healthcare payers. Third, we did not review the gray literature, including reports and websites of agencies known to be associated with payer decision-making and coverage decisions. Although these may be useful sources of information, they were beyond the scope of this review. Fourth, this review did not differentiate between RWE for medical devices and medicines, as few studies on medical devices were identified in the scoping review to support such a distinction. It is acknowledged that RWE may be handled differently for each type of health technology, and so this can be addressed in future research when more relevant studies become available.

## Conclusions

This scoping review highlighted the growing interest in RWE among payers to inform funding and reimbursement decisions for health technologies, particularly for cancer drugs and other pharmaceuticals. RWE provides evidence on long-term clinical effectiveness and safety, real-world cost-effectiveness, and budget impact as well as complements evidence generated from RCTs. Although RWE is more generalizable than evidence generated from RCTs, it is often subject to bias and confounding due to poor study designs and methods, which limit its use in payer decision-making. Managed entry agreements are used by payers and manufacturers to allow the entry of health technologies on the market while allowing for the generation and collection of evidence on a technology’s performance in a clinical setting to inform future reimbursement decisions. They also help to mitigate the risks borne by payers and manufacturers when the clinical and cost-effectiveness of a new technology remains uncertain. Examples of how these agreements function are in contractual arrangements between payers and manufacturers, which are seldom available to the public. Access to these agreements can help researchers better understand how RWE informs reimbursement decisions for new health technologies.

## Supporting information

Masucci et al. supplementary material Masucci et al. supplementary material

## Supplementary material

The supplementary material for this article can be found at http://doi.org/10.1017/S0266462325100445.
