# Supplementary material for: The use of real-world evidence among healthcare payers: a scoping review
Source: Int J Technol Assess Health Care. 2025 Sep 12;41(1):e67. doi: 10.1017/S0266462325100445 (PMC12450548; doi:10.1017/S0266462325100445)
Supplement: Masucci et al. supplementary material [file S0266462325100445sup001.docx]

Supplementary File 1

S1. Search strategy

**PubMed(MEDLINE):**

(RWE[tiab] OR RWD[tiab] OR “real-world”[tiab] OR “big data”[tiab] OR registries[mh] OR registry[tiab] OR registrie*[tiab] OR “population register*”[tiab] OR electronic health records[mh] OR “electronic health record*”[tiab] OR “electronic medical record*”[tiab] OR EHR[tiab] OR EMR[tiab] OR EHRs[tiab] OR EMRs[tiab] OR “online medical record*”[tiab] OR “online health record*”[tiab] OR “national database*”[tiab] OR “clinical data repositor*”[tiab] OR routinely collected health data[mh] OR “claims data*”[tiab] OR “insurance claim*”[tiab] OR “administrative data*”[tiab] OR “hospital data*”[tiab] OR “non-RCT”[tiab] OR “non-randomized controlled trial*”[tiab] OR “non-randomised controlled trial*”[tiab] OR “non-randomized clinical trial*”[tiab] OR “non-randomised clinical trial*”[tiab] OR pragmatic clinical trials as topic[mh] OR “pragmatic trial*”[tiab] OR “pragmatic clinical trial*”[tiab]) **AND**

(single-payer system[mh] OR payer*[tiab] OR payor*[tiab] OR financing, government[mh] OR government agencies[mh] OR “government agenc*”[tiab] OR insurance, health[mh] OR “health benefit plan*”[tiab] OR “health insurance”[tiab] OR “healthcare insurance”[tiab] OR “health care insurance”[tiab] OR “prepaid health care”[tiab] OR “prepaid healthcare”[tiab] OR “medical insurance”[tiab] OR “universal health coverage”[tiab] OR “universal health care”[tiab] OR “universal healthcare”[tiab] OR “reimbursement agenc*”[tiab] OR “reimbursement advisory committee*”[tiab] OR regulator*[tiab] OR “Ministry of Health”[tiab] OR “Minister of Health”[tiab] OR “Ministry for Health”[tiab] OR “Pharmaceutical Benefits Pricing Authority”[tiab] OR “Pharmaceutical Benefits Board”[tiab] OR Medicare[tiab] OR “National Healthcare System”[tiab] OR “Unified Health System”[tiab] OR “National Health Surveillance Agency”[tiab] OR “Patented Medicine Prices Review Board*”[tiab] OR “State Medical Insurance”[tiab] OR “National Health Commission”[tiab] OR “National Fund for Health Insurance”[tiab] OR “National Health Authority”[tiab] OR “National Health Service”[tiab] OR NHS[tiab] OR “National Health Insurance”[tiab] OR “Popular Health Insurance”[tiab] OR “Secretariat of Health”[tiab] OR “Universal Health Insurance”[tiab] OR “General Health Insurance”[tiab] OR “National Institute for Health and Disability Insurance”[tiab] OR “Health Insurance Fund”[tiab]  OR “General Healthcare System”[tiab] OR “General Health Insurance”[tiab] OR “National Healthcare Service”[tiab] OR “National Health System”[tiab] OR “National Organisation for Healthcare Services Provision”[tiab] OR “Health Service Executive”[tiab] OR “National Health Fund”[tiab] OR “National Healthcare Plan”[tiab]) **AND** (decision making, organizational[mh] OR decision making[mh] OR decision support techniques[mh] OR decision*[tiab] OR decid*[tiab] OR judgement[tiab] OR judgment[tiab] OR choice*[tiab] OR negotiat*[tiab] OR accept*[tiab] OR reject*[tiab] OR refuse[tiab] OR refusal[tiab] OR deliberat*[tiab] OR policy making[mh] OR “policy making”[tiab:~3] OR “making policies”[tiab:~3] OR “make policies”[tiab:~3] OR “policy maker*”[tiab] OR “policy development”[tiab:~3] OR “policy developments”[tiab:~3] OR “developing policies”[tiab:~3] OR “policy developed”[tiab:~3] OR “policies developed”[tiab:~3] OR “policy analysis”[tiab:~3] OR “policies analysis”[tiab:~3] OR “policy analyses”[tiab:~3] OR “policies analyses”[tiab:~3]) **AND** English[lang] **AND** 2014:2023[edat]

**Ovid Embase <1974 to 2025 March 31>**

1 exp big data/ 6084

2 exp register/ or exp population register/ 193570

3 exp electronic health record/ 40494

4 exp clinical data repository/ 1433

5 exp routinely collected health data/ 200

6 exp pragmatic trial/ 2292

7 (RWE or RWD or "real-world" or "big data" or registry or registrie* or "population register*" or "electronic health record*" or "electronic medical record*" or EHR or EMR or EHRs or EMRs or "online medical record*" or "online health record*" or "national database*" or "clinical data repositor*" or "routinely collected health data" or "claims data*" or "insurance claim*" or "administrative data*" or "hospital data*" or "non-RCT" or "non-randomized controlled trial*" or "non-randomised controlled trial*" or "non-randomized clinical trial*" or "non-randomised clinical trial*" or "pragmatic trial*" or "pragmatic clinical trial*").ti,ab. 611093

8 or/1-7 679178

9 exp health insurance/ or exp medicare/ or exp national health insurance/ or exp universal health insurance/ 323076

10 exp public finance/ 190

11 exp reimbursement/ and exp advisory committee/ 268

12 (payer* or payor* or "government agenc*" or "health insurance" or "health benefit plan*" or "healthcare insurance" or "health care insurance" or "prepaid health care" or "prepaid healthcare" or "medical insurance" or "universal health coverage" or "universal health care" or "universal healthcare" or "reimbursement agenc*" or "reimbursement advisory committee*" or regulator* or "Ministry of Health" or "Minister of Health" or "Ministry for Health" or "Pharmaceutical Benefits Pricing Authority" or "Pharmaceutical Benefits Board" or Medicare or "National Healthcare System" or "Unified Health System" or "National Health Surveillance Agency" or "Patented Medicine Prices Review Board*" or "State Medical Insurance" or "National Health Commission" or "National Fund for Health Insurance" or "National Health Authority" or "National Health Service" or NHS or "National Health Insurance" or "Popular Health Insurance" or "Secretariat of Health" or "Universal Health Insurance" or "General Health Insurance" or "National Institute for Health and Disability Insurance" or "Health Insurance Fund" or "General Healthcare System" or "General Health Insurance" or "National Healthcare Service" or "National Health System" or "National Organisation for Healthcare Services Provision" or "Health Service Executive" or "National Health Fund" or "National Healthcare Plan").ti,ab. 1203943

13 or/9-12 1395087

14 exp organizational decision making/ 80

15 exp decision making/ 466786

16 exp health care policy/ 224362

17 (decision* or decid* or judgement or judgment or choice* or negotiat* or accept* or reject* or refuse or refusal or deliberat*).ti,ab. 2260221

18 ((policy or policies) adj3 (maker* or making or develop* or analys*)).ti,ab. 70512

19 or/14-18 2667167

20 8 and 13 and 19 12031

21 (conference abstract or conference review).pt. 4884174

22 20 not 21 7580

23 limit 22 to (english language and yr="2014 -Current") 5399

**Cochrane Library:**

#1 (RWE:ti,ab OR RWD:ti,ab OR "real-world":ti,ab OR "big data":ti,ab OR [mh registries] OR registry:ti,ab OR registrie*:ti,ab OR (population NEXT register*):ti,ab OR [mh "electronic health records"] OR (electronic NEXT health NEXT record*):ti,ab OR (electronic NEXT medical NEXT record*):ti,ab OR EHR:ti,ab OR EMR:ti,ab OR EHRs:ti,ab OR EMRs:ti,ab OR (online NEXT medical NEXT record*):ti,ab OR (online NEXT health NEXT record*):ti,ab OR (national NEXT database*):ti,ab OR (clinical NEXT data NEXT repositor*):ti,ab OR [mh "routinely collected health data"] OR (claims NEXT data*):ti,ab OR (insurance NEXT claim*):ti,ab OR (administrative NEXT data*):ti,ab OR (hospital NEXT data*):ti,ab OR "non-RCT":ti,ab OR (non NEXT randomized NEXT controlled NEXT trial*):ti,ab OR (non NEXT randomised NEXT controlled NEXT trial*):ti,ab OR (non NEXT randomized NEXT clinical NEXT trial*):ti,ab OR (non NEXT randomised NEXT clinical NEXT trial*):ti,ab OR [mh "pragmatic clinical trials as topic"] OR (pragmatic NEXT trial*):ti,ab OR (pragmatic NEXT clinical NEXT trial*):ti,ab)

#2 ([mh "single-payer system"] OR payer*:ti,ab OR payor*:ti,ab OR [mh "financing, government"] OR [mh "government agencies"] OR (government NEXT agenc*):ti,ab OR [mh "insurance, health"] OR (health NEXT benefit NEXT plan*):ti,ab OR "health insurance":ti,ab OR "healthcare insurance":ti,ab OR "health care insurance":ti,ab OR "prepaid health care":ti,ab OR "prepaid healthcare":ti,ab OR "medical insurance":ti,ab OR "universal health coverage":ti,ab OR "universal health care":ti,ab OR "universal healthcare":ti,ab OR (reimbursement NEXT agenc*):ti,ab OR (reimbursement NEXT advisory NEXT committee*):ti,ab OR regulator*:ti,ab OR "Ministry of Health":ti,ab OR "Minister of Health":ti,ab OR "Ministry for Health":ti,ab OR "Pharmaceutical Benefits Pricing Authority":ti,ab OR "Pharmaceutical Benefits Board":ti,ab OR Medicare:ti,ab OR "National Healthcare System":ti,ab OR "Unified Health System":ti,ab OR "National Health Surveillance Agency":ti,ab OR (Patented NEXT Medicine NEXT Prices NEXT Review NEXT Board*):ti,ab OR "State Medical Insurance":ti,ab OR "National Health Commission":ti,ab OR "National Fund for Health Insurance":ti,ab OR "National Health Authority":ti,ab OR "National Health Service":ti,ab OR NHS:ti,ab OR "National Health Insurance":ti,ab OR "Popular Health Insurance":ti,ab OR "Secretariat of Health":ti,ab OR "Universal Health Insurance":ti,ab OR "General Health Insurance":ti,ab OR "National Institute for Health and Disability Insurance":ti,ab OR "Health Insurance Fund":ti,ab OR "General Healthcare System":ti,ab OR "General Health Insurance":ti,ab OR "National Healthcare Service":ti,ab OR "National Health System":ti,ab OR "National Organisation for Healthcare Services Provision":ti,ab OR "Health Service Executive":ti,ab OR "National Health Fund":ti,ab OR "National Healthcare Plan":ti,ab)

#3 ([mh "decision making, organizational"] OR [mh "decision making"] OR [mh "policy making"])

#4 (decision*:ti,ab OR decid*:ti,ab OR judgement:ti,ab OR judgment:ti,ab OR choice*:ti,ab OR negotiat*:ti,ab OR accept*:ti,ab OR reject*:ti,ab OR refuse:ti,ab OR refusal:ti,ab OR deliberat*:ti,ab)

#5 (policy:ti,ab OR policies:ti,ab) NEAR/3 (maker*:ti,ab OR making:ti,ab OR develop*:ti,ab OR analys*:ti,ab)

#6 #3 OR #4 OR #5

#7 #1 AND #2 AND #6

Limited to 2014-2025, English language.

**Proquest Dissertations & Theses Global:**

noft((RWE OR RWD OR "real-world" OR "big data" OR registry OR registrie* OR "population register*" OR "electronic health record*" OR "electronic medical record*" OR EHR OR EMR OR EHRs OR EMRs OR "online medical record*" OR "online health record*" OR "national database*" OR "clinical data repositor*" OR "routinely collected health data" OR "claims data*" OR "insurance claim*" OR "administrative data*" OR "hospital data*" OR "non-RCT" OR "non-randomized controlled trial*" OR "non-randomised controlled trial*" OR "non-randomized clinical trial*" OR "non-randomised clinical trial*" OR "pragmatic trial*" OR "pragmatic clinical trial*")) AND noft((payer* OR payor* OR "government agenc*" OR "health insurance" OR "health benefit plan*" OR "healthcare insurance" OR "health care insurance" OR "prepaid health care" OR "prepaid healthcare" OR "medical insurance" OR "universal health coverage" OR "universal health care" OR "universal healthcare" OR "reimbursement agenc*" OR "reimbursement advisory committee*" OR regulator* OR "Ministry of Health" OR "Minister of Health" OR "Ministry for Health" OR "Pharmaceutical Benefits Pricing Authority" OR "Pharmaceutical Benefits Board" OR Medicare OR "National Healthcare System" OR "Unified Health System" OR "National Health Surveillance Agency" OR "Patented Medicine Prices Review Board*" OR "State Medical Insurance" OR "National Health Commission" OR "National Fund for Health Insurance" OR "National Health Authority" OR "National Health Service" OR NHS OR "National Health Insurance" OR "Popular Health Insurance" OR "Secretariat of Health" OR "Universal Health Insurance" OR "General Health Insurance" OR "National Institute for Health and Disability Insurance" OR "Health Insurance Fund" OR "General Healthcare System" OR "General Health Insurance" OR "National Healthcare Service" OR "National Health System" OR "National Organisation for Healthcare Services Provision" OR "Health Service Executive" OR "National Health Fund" OR "National Healthcare Plan")) AND noft((decision* OR decid* OR judgement OR judgment OR choice* OR negotiat* OR accept* OR reject* OR refuse OR refusal OR deliberat* OR policy OR policies))

Additional limits - Date: From January 01 2014 to March 31 2025; Language: English

Studies from databases/registers **(n = 13655)**

Embase (n = 6887)

PubMed (n = 5159)

Proquest Dissertations and Theses Global (n = 832)

CENTRAL (n = 777)

References from other sources **(n = 0)**

Citation searching (n = 0)

Grey literature (n = 0)

**Identification**

Studies excluded **(n = 9584)**

Studies not retrieved **(n = 0)**

Studies assessed for eligibility **(n = 47)**

Studies sought for retrieval **(n = 47)**

Studies screened **(n = 9631)**

Studies excluded **(n = 28)**

Did not meet the inclusion criteria **(n = 28)**

References removed **(n = 4024)**

Duplicates identified manually (n = 3)

Duplicates identified by Covidence (n = 4021)

Marked as ineligible by automation tools (n = 0)

Other reasons (n = 0)

**Screening**

**Included**

Studies included in review **(n = 19)**

Supplementary Figure S1 PRISMA diagram showing selection of included studies (32)

Supplementary Table S1 Data extraction: country of focus, objective, intended audience, disease area, methods, definition of real-world evidence

| **Author(s)/date** | **Country of focus (general/global focus, specific country, please specify, not specified).** | **Objective** | **Intended audience/user (academics, HTA bodies, payers, other, please specify)** | **Application/Disease area** | **Methods/type of paper (primary research: case study, literature review, qualitative study, mixed methods, other, please specify. Other types of papers (commentary, discussion, website content).** | **Definition of real-world evidence** |
| --- | --- | --- | --- | --- | --- | --- |
| Abu-Shraie et al 2023 (41) | Saudi Arabia | To evaluate the current status and impacts of implementing RSAs in KSA, to discuss future applications and anticipated implementation challenges, and to provide examples of RSAs implemented in different sectors in KSA, as well as recommendations on how to implement such agreements in KSA | Payers and health technology manufacturers | Pharmaceuticals | Narrative review | N/A |
| Bharmal et al 2024 (42) | France, USA, UK, Germany, Spain | To confirm whether efficacy and safety results from randomized controlled trials are reflected in real-world outcomes and to confirm the projected utilization of an oncology therapy. | Payers | Oncology | Qualitative: survey | The FDA defines real-world evidence (RWE) as clinical evidence about the usage and potential benefits or risks of a medical product derived from analyses of RWD [1]. |
| Brandes et al 2016 (39) | Germany | This study assesses the use of routinely collected claims data for managed entry agreements (MEA) in the illustrative context of German statutory health insurance (SHI) funds. This study assesses to what extent the claims data of health insurance funds are suitable sources of evidence for different types of MEA. | Coverage decision-makers | New health technologies | Non-systematic review | N/A |
| Brixner et al 2021 (36) | USA | To assess US payer perceptions regarding the use and relevance of RWE in informing oncology formulary decision-making | Payers | Oncology | Mixed methods | N/A |
| Chan et al 2020 (3) | Canada | To describe the CanREValue collaboration and its work toward integrating RWE into cancer drug funding decision-making in Canada | Drug funding decision-makers | Oncology | Description of working groups | RWE, generated through the analysis of real-world data (RWD), can provide direct and generalizable evidence on the comparative effectiveness, safety, and cost-effectiveness of cancer drugs in clinical practice |
| Clausen et al 2020 (6) | Canada | To explore stakeholder perspectives on the current state of RWE in Canada to inform a Canadian framework for the use of RWE in cancer drug funding decisions | Canadian and international stakeholders who had experience with RWE and drug funding decision-making | Oncology | Qualitative descriptive | Real-world evidence (RWE) — evidence from post-market evaluations not derived from traditional RCTs |
| Gray & Kenney 2019 (28) | USA | To identify the necessary conditions to support the widespread adoption of outcomes-based contracts for high-cost drug therapy, with a focus on disease-modifying therapies for multiple sclerosis | Payers, manufacturers, and industry consultants | Multiple sclerosis | Qualitative | N/A |
| Hampson et al 2018 (20) | USA | To explore current uses of real-world evidence (RWE) in the US healthcare system, summarize key concerns, and highlight various opportunities that could be realized through the best use of RWE | N/A | Not specified | Qualitative | RWE is the clinical evidence regarding the usage, and potential benefits or risks, of a medical product derived from analysis of RWD |
| Husereau et al 2019 (37) | Canada | To reflect on perceptions of the value of real-world evidence in pricing and reimbursement decisions, barriers to its optimal use in pricing and reimbursement, current initiatives that may lead to its increased use, and what role the pharmaceutical industry may play | Payers, funding decision-makers | Not specified | Qualitative | Payers and HTA representatives often equated evidence from real-world data with “observational studies” |
| Kirwin et al 2022 (11) | General | We propose the life-cycle (LC)-HTA framework, providing additional tools to decision-makers and improving outcomes for all stakeholders | HTA agencies, patients, payers, and sponsors | Not specified | Qualitative/Description of framework | The result of analyzed administrative, or routinely collected data |
| Kovács et al 2022 (12) | Central and Eastern European countries | To put forward recommendations on how Central and Eastern European (CEE) health technology assessment bodies and payer organizations can apply coverage with evidence development (CED) to reduce decision uncertainty on reimbursement of medical devices, with a particular focus on transferring the structure and data from CED schemes in early technology adopter countries in Western Europe | HTA agencies and payers | Medical devices | Qualitative: decision-making framework | N/A |
| Laba et al 2020 (2) | Canada | To describe the implementation of multi-criteria decision analysis (MCDA) into a Canadian public drug reimbursement decision-making process, identifying the aspects of the MCDA approach, and the context that promoted uptake | HTA agencies and payers | Pharmaceuticals | Qualitative: decision-making framework | N/A |
| Lau & Dranitsaris 2022 (15) | Canada | This article examines reimbursement recommendations by the Canadian Agency for Drugs and Technology in Health (CADTH) on oncology drugs approved between 2019 and 2021 | Regulatory bodies, HTA agencies, payers | Oncology | Quantitative | Real-world evidence (RWE) in medicine refers to evidence obtained from real-world data (RWD), which are health and outcome data generated from a patient’s journey outside the context of controlled clinical trials. RWE is generated by analyzing data obtained from patient registries, medical records, claims databases, or in some cases hybrid trials, pragmatic trials, and late-phase trials |
| Mohseninejad et al 2015 (38) | The Netherlands | This study aimed to develop a model for the regular evaluation of patient registries during an access with the evidence development process and find the the optimal length of the registry period | HTA agencies & payers | Oncology | Quantitative | Registries are a source of RWD/RWE |
| Pearson et al 2018 (43) | General | To provide a framework for optimizing the development and use of real-world evidence (RWE) in drug coverage decisions | Payers, patient groups & manufacturers | Pharmaceuticals | Qualitative: descriptive | Real-world evidence (RWE) – evidence derived from the analysis of data collected either prospectively or retrospectively from routine clinical practice – is transforming the process through which healthcare payers make coverage and formulary decisions for pharmaceuticals |
| Pulini et al 2021 (7) | France, USA, UK | This review aims to illustrate how health authorities in France, the United States (USA), and the United Kingdom (UK) can integrate RWD and RWE in market authorization discussions and in new pathways of price and reimbursement negotiations | Medicine developers, payers, HTA agencies | Pharmaceuticals | Qualitative: non-systematic review | Real-world evidence (RWE) refers to the evidence derived from the analysis and/or synthesis of RWD. RWD are an umbrella term for a variety of data types concerning the effects of health interventions that are not collected in the context of highly controlled, idealized conditions, of RCTs” for “decision-making” |
| Roberts & Ferguson 2020 (8) | USA | We review how payers use or can use RWD on the comparative effectiveness and safety of treatments, PROs, medication adherence and persistence, prescribing patterns, healthcare resource utilization, and patient characteristics and/or biomarkers associated with treatment response when making health technology assessments and payer coverage decisions across therapeutic areas | Payers | Various therapeutic areas | Qualitative: broad literature review | Real-world evidence (RWE) is obtained from an analysis of real-world data (RWD) from prospective or retrospective sources, including electronic health records, claims databases, pragmatic trials, or registries, but they may also be gathered from patient-generated sources such as smartphones, wearable devices, and survey data |
| Timbie et al 2021 (17) | USA | We described the current use of RWE for medical devices, assessed manufacturers’ challenges in generating and using it for regulatory and coverage decisions, and identified opportunities to expand its use. | Manufacturers & payers | Medical devices | Descriptive | Evidence, which is generated outside of research settings |
| Xoxi et al 2022 (40) | Italy | The aim of this project is to propose a new pathway of value-based MEA (VBMEA), based on the analysis of the current Italian pricing and reimbursement framework. | Payers, HTA, medical product manufacturers | Not specified | Descriptive: framework with non-systematic review |  |

RSA – risk-sharing agreements; KSA – Kingdom of Saudi Arabia; N/A - not applicable; RWE – real-word evidence; CanREValue - Canadian Real-world Evidence for Value of Cancer Drugs; RCTs – randomized controlled trials; HTA – Health technology assessment; LC-HTA-Lifecycle health technology assessment

Supplementary Table S2 Data extraction: Reasons, strengths and weaknesses of real-world evidence, and types and use of real-world evidence

| Author(s)/date | **Reason for using real-world evidence** | **Strengths and weaknesses of real-world evidence as described in the paper.** | **What type of real-world data was discussed (clinical, economic, patient preferences (utilities), other, please specify)** | **How was RWE used by healthcare payers? Please specify** | **Were any performance-based reimbursement schemes discussed (no, yes, if yes, what reimbursement scheme: coverage with evidence development, conditional treatment continuation, performance-based reimbursement, other, please describe)** |
| --- | --- | --- | --- | --- | --- |
| Abu-Shraie et al 2023 (41) | N/A | N/A | N/A | N/A | Risk-sharing agreement: outcome-based and financial-based arrangements |
| Bharmal et al 2024 (42) | To confirm whether efficacy and safety results from randomized controlled trials are reflected in real-world outcomes and to confirm the projected utilization of an oncology therapy. | N/A | Prospective observational studies, product/disease registries | N/A | N/A |
| Brandes et al 2016 (39) | N/A | N/A | Claims data from registries | N/A | **Managed entry schemes:** Health outcomes- coverage only in research, coverage with evidence development, conditional treatment continuation, outcomes-based risk sharing; Utilization & Cost -utilization-based risk sharing, cost-based risk sharing |
| Brixner et al 2021 (36) | N/A | N/A | Claims data, medical records, patient registries, prospective cohort studies, patient outcome surveys | To expand the understanding of how payers in the US use RWE to inform healthcare decision-making in oncology. This information can help payers identify and prioritize relevant RWE from researchers and help manufacturers design RWE studies that would be relevant to payers | Outcome-based contracting |
| Chan et al 2020 (3) | N/A | **Strengths:** external validity; less expensive. **Limitations:** bias, confounding | N/A | N/A | N/A |
| Clausen et al 2020 (6) | To provide information on clinical effectiveness, safety, cost-effectiveness and budget impact outside of the highly controlled trial environment to determine whether an intervention yielded expected outcomes | **Strengths**: post-market decision; **Limitations:** bias, confounding, inconsistent data collection, analytic methods and conclusions. | N/A | N/A | N/A |
| Gray & Kenney 2019 (28) | N/A | N/A | N/A | N/A | Outcomes-based contracting |
| Hampson et al 2018 (20) | N/A | **Strengths:** drug development, regulatory approval decision, post-approval monitoring of safety signals, HTA & payer coverage decision-making **Limitations:** bias & confounding, incomplete data, data mining, access to data, lack of universally accepted methodological standards, lack of investigator expertise, obsolete evidence hierarchies | N/A | N/A | Outcomes-based contracting |
| Husereau et al 2019 (37) | N/A | **Barriers to use of RWD:** Access to data, governance, data quality, and comparability, standard in interpretation, type of data collected, human resources | N/A | N/A | N/A |
| Kirwin et al 2022 (11) | N/A | N/A | N/A | LC-HTA | Research-oriented managed access agreement, risk-based pricing |
| Kovács et al 2022 (12) | N/A | N/A | N/A | N/A | Coverage with evidence development |
| Laba et al 2020 (2) | N/A | N/A | N/A | N/A | N/A |
| Lau & Dranitsaris 2022 (15) | N/A | N/A | N/A | N/A | N/A |
| Mohseninejad et al 2015 (38) | To evaluate the use of registry data to support the access with evidence process for the reimbursement of oxaliplatin for stage III colon cancer treatment | **Strengths:** reflect daily practice more closely and can include a larger population than randomized studies **Limitations**: risk of biased outcomes | N/A | N/A | N/A |
| Pearson et al 2018 (43) | N/A | **Limitation:** relevance, timeliness and transparency | N/A | N/A | N/A |
| Pulini et al 2021 (7) | N/A | **Limitations:** lack of transparent and standardized outcome data; privacy, protection of individual data and consent issues, data validity and quality, capability and capacity in data extraction (technology and methodology), clarity on the acceptability of RWE, and guidance from regulators | N/A | N/A | N/A |
| Roberts & Ferguson 2020 (8) | N/A | N/A | N/A | N/A | N/A |
| Timbie et al 2021 (17) | N/A | N/A | N/A | N/A | N/A |
| Xoxi et al 2022 (40) | To integrate with evidence from RCTs to confirm drug benefits & risks and inform risk-sharing agreements | N/A | N/A | N/A | Value-based managed entry agreement |

RWE – real-world evidence, N/A - not applicable; HTA – health technology assessment, RCTs – randomized controlled trial.
